# Perioperative Factors Affecting the Healing of Rectovaginal Fistula

**DOI:** 10.3390/jcm12196421

**Published:** 2023-10-09

**Authors:** Małgorzata Satora, Klaudia Żak, Karolina Frankowska, Marcin Misiek, Rafał Tarkowski, Marcin Bobiński

**Affiliations:** 1 I Chair and Department of Oncological Gynaecology and Gynaecology, Student Scientific Association, Medical University of Lublin, 20-081 Lublin, Poland; msatoraa@gmail.com (M.S.); zakklaudia3@gmail.com (K.Ż.); k.frankowska10@gmail.com (K.F.); 2 Department of Gynecology, Holy Cross Cancer Center, 25-734 Kielce, Poland; mmisiek@me.com; 3 I Chair and Department of Oncological Gynaecology and Gynaecology, Medical University of Lublin, 20-081 Lublin, Poland; rafaltar@yahoo.com

**Keywords:** rectovaginal fistula, healing, preoperative factors

## Abstract

Rectovaginal fistula is rare, but a severe complication in gynecology, which despite the effort of clinicians is still not treated successfully in many cases. According to statistics, the healing rates of surgery in patients with RVF range from 20 to 100%. The treatment effectiveness depends on the etiology of fistula, the age of the patients, the presence of comorbidities, the type of surgery and many other factors. Considering the low efficiency of treatment and the high risk of recurrence, the question of possible methods to improve the results occurs. In our review, we analyzed both modifiable and non-modifiable factors which may influence the treatment, healing rate and future fate of the patients. Taking into account all analyzed risk factors, including age, comorbidities, smoking status, microbiology, medications, stoma and stool features, we are aware that rectovaginal fistula’s treatment must be individualized and holistic. In cases of poorly healing RVF, the drainage of feces, the use of antibiotic prophylaxis or the implementation of estrogen therapy may be useful. Moreover, microbiome research in women with RVF and towards estrogen therapy should be performed in order to create treatment algorithms in women with fistulae. Those interventions, in our opinion, may significantly improve the outcome of the patients.

## 1. Introduction

Despite the efforts of clinicians, the treatment of rectovaginal fistula (RVF) is still one of the challenges for physicians, especially gynecologists and surgeons. Depending on statistics, the healing rates of surgery in RVF range from 20 to 100% [1], which is still not satisfying. The authors of the studies regarding the main cause of unsatisfactory treatment results see the presence of persistent inflammation, which triggers a prolonged wound healing and as a consequence the excessive deposition of extracellular matrix further hindering recovery [2,3]. Therefore, the frequency of RVF recurrences is high and ranges from 20% to even 90% [4,5,6,7].

Why is this an issue? First of all, we should not forget that RVF is a miserable disease, which causes psychological disorders: above all, fear or depression [8]. The influence of RVF on the quality of life has been presented in many studies. Leroy et al. showed that preoperatively, 50% of patients with fistula report anxiety in comparison to 0% postoperatively. Moreover, in the study, patients with fistulas had a decreased quality of life—in the social and sports domains [9]. Söderqvist et al. showed that patients with an unhealed fistula had lower scores in 6 out of 8 domains of the Health-Related Quality of Life questionnaire than both healed patients and the normal population [3]. Secondly, sexual dysfunctions occur. In the study of Kazi et al., only 4 of 488 patients (0.8%) were sexually active [10]. Thirdly, among all domains of global disability status, everyday activities and socializing are the most affected [11]. Women with RVF are afraid of going out and stay in their houses most of the time.

Taking into consideration the negative impact of RVF on the quality of life, sexual functions, psychological disorders and unsatisfactory treatment results, it seems necessary to ask ourselves if it is possible to increase the efficacy of its treatment. Therefore, the aim of this study was to identify the perioperative factors influencing the healing rate including comorbidities, preoperative interventions like antibiotic prophylaxis, protective stoma, the microbiome and many others. Additionally, our goal was to find methods which could improve the results of the available treatment methods.

## 2. Materials and Methods

The purpose of this article was to describe perioperative factors affecting healing and repairing RVF. The review of the scientific literature was carried out without a time limit until March 2023 using PubMed, Google Scholar and Science Direct databases.

**Figure 1:**
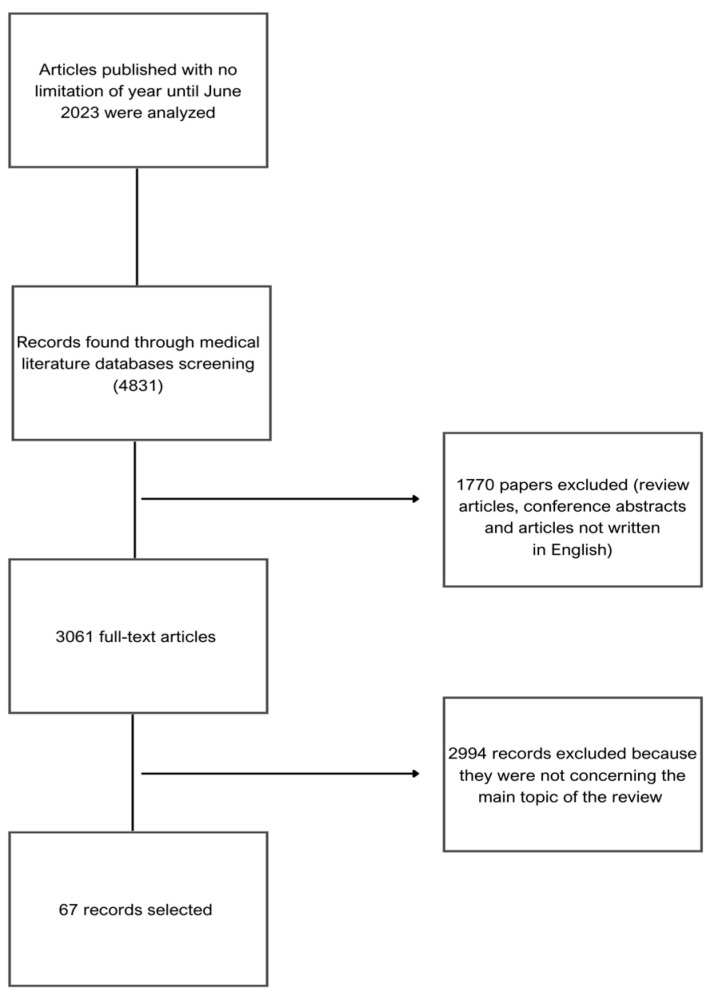
Search criteria for articles used for this review.

Keywords such as “rectovaginal fistula” were used together with “Perioperative Factors”, “Crohn’s disease”, “Cancer”, Age”, “Obstetric”, “Microbiota”, “Antibiotic”, “Treatment”, “Smoking”, “Comorbidities” and “Stoma” to find articles that meet the goals of the review. The inclusion criteria for the papers were the following: original papers, retrospective studies and clinical cases related to RVF. Exclusion criteria were review articles, articles not written in English and duplicated papers. Additionally, abstracts from conferences and articles out of the subject of the review were also excluded. The detailed selection of literature is described in Figure 1.

## 3. Etiology of RVF

In the literature, RVF is divided according to etiology, most often including obstetric and non-obstetric fistulas. Obstetric complications are the most common etiology of traumatic RVF (88%) and include third- and fourth-degree lacerations during vaginal delivery [12]. Non-obstetric RVF is most commonly caused by CD (Crohn’s Disease) (2.1%), iatrogenic trauma, malignancy, radiation, or non-surgical trauma and foreign bodies [13]. It seems that the etiology of RVF will be important in the selection of the method of treatment and the results of surgical treatment of the fistula. Taking into account primarily the etiology of RVF, as well as its size and location, the Rothenberg classification distinguishes simple and complex fistulas. Simple RVFs are low, small and arise as a result of infection or mechanical trauma (RVF resulting from obstetric complications). In turn, complex RVFs are large, high and result from cancer, radiotherapy, inflammatory diseases or diseases of the large intestine [14]. The choice of the surgical method of RVF treatment is influenced by this classification, the history of surgeries, the integrity of the anal sphincters and the quality of the surrounding tissues, which are often dependent on the cause of the fistula formation [15].

In a 2019 paper, Karp et al. evaluated the results of RVF treatment resulting from obstetric injuries. A total of 88 women participated in the study, including 53 patients with obstetric RVF and 35 patients with non-obstetric RVF (11 for inflammatory bowel disease (IBD), 4 traumatic, 10 iatrogenic, 7 unknown and 3 other). In addition, the mean age of patients with non-obstetric RVF (53.0) was significantly higher than that of patients with obstetric RVF (37.0). The RVF repair failure rate was 11.3% for obstetric RVFs and 50.0% for non-obstetric RVFs. An analysis of the factors associated with repair failure therefore showed that non-obstetric etiology increases the likelihood of RVF repair failure [16]. Perhaps the reasons for better repair results in obstetric RVFs should also be sought in the lower average age of patients, and thus in the greater influence of estrogens on vaginal trophism; therefore, these two factors are discussed in further chapters of this paper.

CD is the second most common cause of RVF, after obstetric complications. The RVF healing rate in patients with CD is approximately 43–58% [17,18,19]. Despite the known etiology of the disease, its effect on fistula healing remains controversial and research on the subject is inconsistent. In a study by de La Poza et al., no influence of the location and clinical presentation of CD on the response to treatment of genital fistulae was observed. However, the limitation of this study was the heterogeneity of the fistula types in the patients—out of 1215 patients with CD, 47 women had a fistula, including 35 with RVF [20]. The factors involved in the pathogenesis of CD are transforming growth factor β, TNF and IL-13 in the inflammatory infiltrate, which induce the epithelial–mesenchymal transition and the upregulation of matrix metalloproteinases, which may lead to the formation of a fistula [21]. Perhaps cytokines, such as IL-12 or IL-13, responsible for the inflammatory response should be a therapeutic target in CD patients with RVF, which would improve fistula healing especially among CD patients. All studies examining the effect of CD on RVF repair are presented in the Table 1.

Cancer RVF can be caused by direct invasion of a rectal, vulvar, cervical or vaginal tumor. Pelvic cancers are often treated with radiotherapy and/or chemotherapy. Radiation causes changes in blood flow, leading to ischemia around the target tissue, resulting in desquamation, erythema, edema, fibrosis and necrosis. Moreover, radiotherapy has a significant impact on the regenerative capacity of the irradiated tissue, because rapidly dividing epithelial and mucosal endothelial cells are radiosensitive. Thus, it seems that due to the loss of the ability of these cells to divide, the tissues around the RVF will heal less well, which will significantly reduce the likelihood of successful RVF repair [22,23]. As early as 1986, Cooke et al. showed a radiation-induced RVF repair failure rate of 93% (55 patients) with abdominal access [24]. In turn, Nowacki et al. in 1991 reported a failure rate of 78.3% in patients with RVF of the same etiology, also operated by abdominal access [25]. Such a high percentage of RVF repair failures as a result of radiotherapy is a reason to look for therapies aimed at increasing the regenerative capacity of irradiated tissues.

There are studies on the effectiveness of using mesenchymal stem cells (MSCs) in regenerative therapies of radiation enteropathies. MSCs secrete growth factors, immune mediators and anti-fibrotic effectors that are involved in the process of tissue regeneration [26,27]. Despite the lack of studies on the effect of MSCs on the treatment of RVF caused by irradiation, the results of other studies may indicate their potential use in the treatment of these fistulas. Lorenzi et al. showed that MSC injection improved muscle recovery and improved anal sphincter function after injury in rats [28]. Moreover, treatment with MSCs can inhibit fistula formation. In a patient with prostate cancer treated with radiotherapy, administration of MSC with 40 mL of his daughter’s bone marrow led to stable remission of the cancer and inhibition of vesico-rectal fistula formation [29]. The use of MSCs may therefore be a promising strategy in the treatment of lesions caused by irradiation and regeneration of tissue damage. This is a premise for conducting clinical trials on the use of MSC in women with RVF.

Summarizing, obstetric RVFs appear to have a higher chance of successful repair due to the young age of the patient. The vast majority of patients with non-obstetric RVF are postmenopausal. In addition, non-obstetric RVF may be more difficult to treat due to changes resulting from active inflammation associated with disease or radiation. Considering the higher risk of failure of repair of non-obstetric RVFs, a multidisciplinary approach to the treatment of fistulas with complicated etiologies seems to be important. The cooperation of specialists may be necessary in stabilizing the patient’s condition and thus improving the quality of the surrounding tissues, which may improve the effectiveness of RVF repair.

## 4. Patient Related Factors

### 4.1. Not Modifiable

#### 4.1.1. Age

It seems that the patient’s age influences the effectiveness of RVF repair. In a 2021 study, Raju et al. compared the perioperative results of transvaginal/perineal and abdominal access in RVF repair. A total of 2288 women were qualified for the study, including 1560 who underwent transvaginal/perineal surgery (TV/P) and 728 operated from the abdominal cavity. It was shown that age was a factor determining the type of surgery. The median age of women undergoing TV/P repair was 46 years, compared to 63 years for women undergoing abdominal surgery. Older patients were more often qualified for abdominal RVF repair, this observation was explained by the authors by the higher incidence of comorbidities in this group. Regarding complications, 26% of women undergoing abdominal RVF experienced serious complications and 9% of women undergoing TV/P RVF had serious complications. The most common complications were bleeding, superficial surgical site infection, urinary tract infection and sepsis [30]. Therefore, the probable cause of poorer fistula repair results in women operated by abdominal access was not only a large number of comorbidities but also an older age. The selection of patients for the abdominal and transvaginal/perineal surgical approach according to age remains controversial. On one hand, the healing process is altered by age and this might affect the closure of transvaginally operated fistulas; on the other, the abdominal approach is much more traumatic and usually associated with a higher rate of complications. It is also crucial to take into account the fact that the healing of RVF will be dependent on blood supply. RVF repair can be failed because of poor vascularization of the surrounding tissues; thus, in each case this parameter has to be taken into account while selecting the repair method [31].

The elderly are predisposed to wound infection or the development of chronic wounds, but the effect of aging on wound healing is not well understood. Cell aging and increased MMP activity may not only prolong inflammation but also reduce the fibrous response, leading to wound weakening after closure. Such factors may therefore contribute to prolonged wound healing and an increased risk of wound chronicity. Difficulties in cell migration to the wound site may be due to abnormal collagen deposition [32]. To our knowledge, there are currently no data on the effect of age on mucosal healing in the context of fistulas. England et al., in 2006, showed that the wounds of the palatal mucosa heal much slower in the elderly compared to younger people, regardless of gender [33].

Perhaps the reasons for the better rate of RVF repair in younger women should be sought in the greater effect of estrogens on vaginal trophism. The key role in the proper functioning of the connective tissue of the vagina is played by estrogens, which act through the estrogen receptor alpha located in the vaginal epithelium. They not only increase the synthesis of collagen and elastin, but also regulate local blood flow and the degree of permeability of the epithelium, which affects its hydration [34]. In addition, estrogens increase the phagocytic activity of macrophages, which may be of key importance in the cellular mechanisms of the immune response [35]. To the best of the authors’ knowledge, the effects of estrogens on RVF healing are still not fully understood, and the only data available are those regarding their effects on other pelvic or vaginal reconstructive procedures. In an updated 2012 study, Karp et al. demonstrated that early intravaginal administration of estrogen after pelvic reconstruction in postmenopausal patients effectively improved postoperative markers of tissue quality [36]. Moreover, Rahn et al. showed that the preoperative vaginal administration of estrogen reduces the activity of degradative enzymes and improves the integrity of the connective tissue, which facilitates the placement of surgical sutures during surgical repair [37]. Therefore, it seems reasonable to conduct further studies evaluating the effect of estrogen administration and the mechanism of their action on tissue healing in elderly patients undergoing vaginal reconstruction, including RVF repair.

It seems, therefore, that older age will not only affect the choice of RVF treatment method but will also be an inherent risk factor for poor wound healing, and thus also the formation of fistulae. Older patients will be operated on more often in the abdominal cavity than younger patients who undergo TV/P repair. Internal changes in the skin and mucous membranes that occur with age cause not only slow wound healing and a slower rate of wound closure but also increase the risk of chronic fistulas. Moreover, the healing effect of RVF appears to be influenced by estrogens affecting vaginal trophism. Perhaps this factor is also the reason for better healing of obstetric RVFs in younger women and the worse healing of non-obstetric RVFs which was described in the previous chapter of this paper. Future studies evaluating the effect of female sex hormones on RVF healing may help to understand the reasons for poorer RVF repair outcomes in postmenopausal patients, as well as to determine the possible implementation of estrogen supplementation in these women.

#### 4.1.2. Comorbidities

The most common comorbidities in women with RVF include obesity, CD, hypertension, diabetes or preoperative infection [38,39,40]. Despite the conflicting data regarding RVF healing in these diseases, it is extremely important to understand the interaction between these comorbidities and potential mechanisms affecting fistula healing. Knowledge of the likely impact of comorbidities on the treatment of fistulae will allow optimization of the treatment of patients with RVF by minimizing complications caused by the diseases. In a 2021 study, Chong et al. examined the relationship between patients’ comorbidities, the RVF repair pathway and the incidence of diseases and other perioperative complications in 1391 women. RVF repair was performed trans-abdominally (Group 1) in 159 women, trans-perineally in 253 (Group 2) and transvaginally/transanally in 979 (Group 3). The highest percentage of comorbidities was in Group 1: 65 patients were obese (40.9%), 20 (12.65%) had diabetes and 69 (43.4%) had hypertension. In Group 2, 103 (40.7%) patients were obese, 17 (6.7%) had diabetes and 52 (20.6%) had hypertension. In turn, in the third group, 387 (39.5%) patients were obese, 74 (7.6%) had diabetes and 209 (21.3%) had hypertension. The patients from the first group had more postoperative complications and RVF recurrences [7]. In turn, in 2021, the study by Frontali et al. suggested no effect of comorbidities on the failure rate of RVF and pouch-vaginal fistula repair. In total, 68 women with fistula, including 51 with RVF, were operated on, of whom 30 patients had CD, 8 patients had ulcerative colitis, 4 patients had cancer and 10 patients had fistula due to obstetric complications. The results of the study showed that the failure rate of RVF repair was 40%, regardless of the etiology of the fistula [17].

To our knowledge, there are currently no data or studies on the effect of hypertension on RVF repair. Given that hypertension may be associated with abnormal skin wound healing, its effect on fistula healing should also be determined. The cause of changes caused by hypertension may be functional changes in the cells forming keloids (pericytes, endothelial cells, mast cells, skin fibroblasts), as well as inflammation and hypoxia, which results in the formation of a pathological scar [41]. Therefore, it cannot be ruled out that patients with RVF and concomitant hypertension may be susceptible to pathological postoperative scar formation or fistula recurrence. Therefore, both pressure control during surgery and maintaining proper pressure in the perioperative period seem to be important factors for scar healing.

Although diabetes is a common comorbidity in patients with RVF, there are still no studies evaluating the effect of hyperglycemia for fistulas repair. Hyperglycemia impairs endothelial cell function as well as protein synthesis and the proliferation of keratinocytes and fibroblasts. Elevated blood glucose levels may result in the production of reactive oxygen species, the increased amount of which adversely affects the later stages of wound healing [42,43,44]. It should also be remembered that in patients with diabetes, one of the side effects of intensive hypoglycemic therapy is hypoglycemia. Its consequence may be the development of inflammation as a result of the increased expression of ICAM, VCAM, E-selectin, VEGF or an increase in the concentration of the pro-inflammatory cytokine IL-6 in serum [45]. In addition, hypoglycemia may cause an increase in tissue plasminogen activator (tPA) and aldosterone, leading to endothelial dysfunction [46]. Frequent, repeated hypoglycemia therefore prolongs inflammation and endothelial dysfunction, and thus is a factor in the development of preclinical atherosclerosis in Type 1 diabetes. In summary, both hyperglycemia and hypoglycemia are important factors in wound healing. Monitoring blood glucose levels and managing diabetes is important not only for the diabetic foot but also for the healing of other wounds. Therefore, it seems important in the context of patients with RVF, in whom proper fistula healing will improve the quality of life and return to normal functioning.

Despite conflicting data regarding RVF healing in the most common comorbidities such as diabetes, obesity and hypertension, it is extremely important to understand the interaction between these conditions and potential mechanisms affecting fistula healing. Before proceeding with RVF repair, it seems necessary to stabilize and treat comorbidities in patients with RVF. Clinical management of metabolic syndrome, which is reported to affect more women than men, also seems important [47]. Despite the lack of information on the impact of comorbidities in women with fistulae, disease control will reduce inflammation in the body and thus possibly the likelihood of successful fistula healing. In addition, the stabilization and treatment of metabolic syndrome may be associated with a reduced risk of RVF recurrence after surgery. An interdisciplinary approach and constant cooperation of the operator, e.g., consultation with an endocrinologist, diabetologist or cardiologist, is therefore essential in the treatment of RVF in patients with chronic diseases. Given the fact that RVF surgical treatment is planned well in advance, good patient preparation by stabilizing comorbidities and chronic conditions may be the key to successful fistula surgery.

### 4.2. Modifiable

#### 4.2.1. Microbiology

It is still unknown how the vaginal and/or rectal microbiome may affect RVF healing. Leach et al. characterized the rectal and vaginal microbiomes of 14 RVF patients. The dominant taxa of the vaginal microbiome in patients were *Lactobacillus* (16.2%), *Bacteroides* (9.4%) and *Prevotella* (6.4%), and the anal microbiome was *Bacteroides* (14.6%), *Parabacteroides* (5.8%), *Prevotella* (5.5%) and *Faecalibacterium* (5.4%) [48]. This is extremely important because the microflora of a healthy patient of reproductive age is dominated mainly by *Lactobacillus crispatus*, *Lactobacillus gasseri*, *Lactobacillus inert* and *Lactobacillus jensenii* [49]. According to the study of Leach et al., the increased diversity of the anal microbiome had an impact on the diversity of the vaginal microbiome, as fecal contamination of the vagina allows other microbial species to grow in the vagina. The study also showed that the vaginal microbiome of a patient with RVF was more similar to the rectal microbiome of the same patient than to the vaginal microbiome of a healthy person. The composition of the vaginal microbiome and pH will vary from patient to patient [48].

Mitalas et al. showed that most rectal fistulas are lined with granulation tissue, inducing an inflammatory response [50]. In 2013, van Onkelen et al. analyzed the influence of bacterial infection on fistula persistence. The main component of the bacterial cell wall is the pro-inflammatory peptidoglycan, which stimulates the processing and secretion of the cytokine interleukin IL-1B [51]. In 2016, scientists proved the expression of this cytokine in 93% of anal fistulas [52]. Perhaps the use of additional staining to identify cytokine-producing cell types would allow for a better understanding of cytokine effects on the inflammatory process in RVF, and thus the initiation of supportive therapy for fistulae by targeting cytokine-producing cells.

*Lactobacillus* are bacterial bacilli that have antimicrobial and anti-inflammatory properties. Women with a less diverse microbiome and higher levels of Lactobacilli are thought to have a lower incidence of bacterial disease. In patients with RVF, the reduced level of *Lactobacillus* may be the cause of increased microbiome diversity and bacterial vaginosis by presence of an “ecological niche” for other species [53,54]. According to a 2021 study, a modulator of RVF surgical success may therefore be modifying the vaginal or anal microbiome to improve the overall condition of the anus or vagina [48]. Probiotics, thanks to their anti-inflammatory and antimicrobial properties, modulate the microflora. Ahmadi et al. showed that the use of probiotics for 12 weeks resulted in a decrease in the BMI of polycystic ovary syndrome (PCOS) patients, as well as a decrease in glycemia and cholesterol [55]. On the other hand, in a meta-analysis, Heshmati et al. showed the influence of probiotics primarily on glycemia and lipid metabolism [56]. Considering the possible adverse effects of hyperglycemia and hypoglycemia on RVF healing described in the previous section, perhaps the use of probiotics in fistula patients would have a positive effect on fistula repair.

Fecal microbiome transplantation (FMT) is a method that enables the introduction of microorganisms from the feces of healthy donors into the recipient’s digestive tract in order to change the intestinal microbiome [57]. In 2013, Zhang et al. described the treatment of FMT in a patient with internal fistula and coexisting refractory CD. A single FMT resulted in clinical remission of the disease for approximately 9 months. Further follow-up of the patient was not described in the study. The study, however, confirms the positive effect of FMT in patients with CD [58]. In their study, Xiang et al. evaluated the effect of FMT on the treatment of 174 patients with CD. The results of the study showed that in 4/5 patients, the enterocutaneous fistula closed. However, the exact numbers of patients with pre-existing perirectal fistula and enterocutaneous fistula are not listed due to the limited sample size. Moreover, previously these patients were treated with exclusive enteral nutrition (EEN). Thus, perhaps the modification of the intestinal microflora with FMT increases the likelihood of closure of enterocutaneous fistulae and may be helpful together with EEN in the treatment of fistulae [59]. Due to the potential capabilities of these two methods of microbiome modification, further research is needed to demonstrate the effectiveness of probiotics and FMT in RVF healing.

Biofilm is a complex matrix that provides protection to microorganisms. To our knowledge, there are no studies describing the role of biofilm in RVF. In a study from 2017, the biofilm in chronic anal fissures (CAF) was analyzed—the thickest biofilm was made by *Escherichia coli* and *Pseudomonas aeruginosa*, and the medium-thick biofilm was made by *Pseudomonas aeruginosa*, *Enterococcus* spp. and *Staphylococcus aureus* [60]. Therefore, it is possible that bacteria producing a thick layer of biofilm will contribute to fistula treatment failure. Moreover, Jaiswal et al. in a 2021 study, described biofilm produced in patients with anal fistulas lasting more than 6 months. This is the first study to measure this microbial aspect of prolonged anal fistulas. As in the previous study, *Estetichia coli* (50%) was the main fistula microorganism in this study. The remaining bacteria are mainly Klebsiella pneumoniae, *Enterococcus* spp. and *Prevotella* spp. The authors made a similar hypothesis that patients with a fistula lasting more than 6 months had intestinal bacteria producing a thick layer of biofilm [61]. While the results of these studies cannot directly determine the contribution of biofilm-forming organisms to the progression of fistula infection, it would seem that in the case of a predominance of medium- to high-level biofilm-producing microflora, anal fistulae would heal poorly [60,61]. Although the study results below apply to CAF and rectal fistulae, the similar microbiome in RVF patients may warrant further research to elucidate the role of biofilm formation in fistula chronicity. Perhaps a thorough understanding of the RVF biofilm and the possibility of its treatment with FMT or probiotics will significantly increase the percentage of cured women in the future and reduce the recurrence of fistulae.

All studies describing the connection between microbiome and anal fistulas are presented in the Table 2.

#### 4.2.2. Smoking

Furthermore, it seems necessary to assess the effect of tobacco use on fistula healing [33]. The available literature indicates undesirable effects of RVF repair in smoking patients. In a study from 2010, the risk of fistula recurrence in patients who smoke cigarettes was 95% [4]. De La Poza et al. identified 47 patients with genital fistula in a study. Never smokers (43.8%) were found to be more likely to close their fistula than current or former smokers (22.1%) [20]. Substances present in cigarettes cause vasoconstriction, greater platelet adhesion and an impaired inflammatory response, which in turn may lead to cell dysfunction and tissue hypoxia [62,63,64]. Moreover, studies show that cigarette smoking has an impact on chronic inflammation and autoimmunity [65,66]. Smoking also affects molecular pathways such as MAP (mitogen-activated protein kinases) kinases, histone modification and NFκB (nuclear factor kappa-light-chain-enhancer of activated B cells) [67]. As for the effect of smoking on anal tissues, already in a 1997 study, Palefsky et al. showed that tobacco could cause anorectal cytological abnormalities at the CD4 level [68]. In 2004, Phillips et al. showed that components of cigarette smoke cause genotoxic damage to the anal epithelium [69]. To our knowledge, there are currently no studies showing a significant relationship between smoking and RVF healing. The 2017 study by Zheng et al. is the first study showing a significant impact of smoking on the development of rectal fistulas. However, from our point of view the biggest limitation of the study is the inclusion of only males in their research [70].

Due to the high risk of fistula formation in women, especially in CD, further clinical trials should be conducted to evaluate the effect of smoking on fistula formation in women. Due to the composition of cigarette smoke and the health status of fistula patients, smoking may impair the immune response, leading to immunodeficiency that may affect RVF healing. Understanding the effect of tobacco on RVF may help clinicians inform smokers of the potential benefits of quitting smoking before surgery. It also seems important to determine the time necessary to improve the patient’s condition after smoking cessation, in order to be able to plan the treatment algorithm for the woman in advance.

## 5. Interventions (Treatment Depended)

### 5.1. Perioperative Antibiotic Therapy and Medications

While there are articles describing the effect of pharmacotherapy on RVF, to the best of our knowledge, there are still no precise guidelines on the use of antibiotics in patients with RVF. According to the guidelines of the American Society of Colorectal Surgeons, non-surgical management with antibiotics in the treatment of RVF may be used mainly in obstetric or minimally symptomatic fistulas [65]. Guidance on the use of antibiotics in other gynecological procedures may be helpful to further determine the effect of antibiotic therapy on RVF repair. According to the 2012 recommendations for antibiotic prophylaxis by the Canadian Infectious Diseases Committee in other gynecological procedures, the implementation of antibiotic therapy may be necessary to combat these diseases or the accompanying inflammation. Antibiotic prophylaxis in gynecology concerns primarily abdominal or transvaginal hysterectomy and other procedures involving opening vagina to the abdominal cavity. Antibiotics are usually given 15–60 min before surgery. It may also seem important to administer an additional dose of antibiotic in the case of long-lasting abdominal surgery or with blood loss of more than 1500 mL. In women with obesity (BMI > 35 kg/m^2^), it is worth considering adjusting the dose of antibiotics. Moreover, in patients undergoing gynecological procedures where antimicrobial prophylaxis is recommended, cephalosporin is usually used in the absence of hypersensitivity to penicillin [49].

Regarding the use of antibiotics in fistula patients, studies have focused on their impact and effects in patients with rectal fistulae and concomitant CD. George et al., in a 2019 study, showed that taking low-dose metronidazole was associated with a reduced risk of malignant fistulas in patients with recurrent cervical cancer. In total, 208 women with locally recurrent cancer were selected for the study, 76 of whom received metronidazole as a maintenance therapy. Fistula developed in 22.4% of patients receiving metronidazole and 41.7% of patients not taking metronidazole [71]. In turn, Brandt et al. described the recurrence of perianal fistula after discontinuation of metronidazole in 10 patients [72].

The probable mechanism of action of metronidazole in the prevention of RVF should be seen in the reduction in anaerobic necrosis, which in the case of cervical cancer may be a risk factor for RVF [71]. Due to its good pharmacokinetic and pharmacodynamic properties, immediate action and effectiveness in preventing necrosis, metronidazole can be used prophylactically in patients to prevent fistulas. Therefore, it seems that the control and implementation of metronidazole treatment in patients with RVF may not only reduce the failure of treatment and poor healing of the fistula but also prevent its formation. Study results indicating a lower risk of fistula formation in patients treated with metronidazole should be carefully considered for the development of a uniform regimen for the use of this antibiotic in patients with (or incipient) development of RVF.

The Enhanced Recovery After Surgery (ERAS) protocol is used to support perioperative care for patients undergoing elective abdominal surgery. Cavallaro and others summarized ERAS at Massachusetts General Hospital and proposed the regimen of patients’ preparation for colon surgery. To reduce the risk of infection, patients should follow the preoperative regimen of intestinal emptying by using laxatives (based on commonly using agents such as: bisacodyl, polyethylene glycol). One hour after bowel preparation, the patient takes a combination of at least two antibiotics (neomycin 1000 mg/erythromycin 500 mg or neomycin 1000 mg/metronidazole 500 mg). Protocols with detailed preoperative management for patients undergoing colon surgeries may be an important premise for the use of similar schemes in other procedures [73].

An interesting approach based on the inflammatory mechanism of fistula formation is to utilize anti-TNF (anti-tumor necrosis factor) agents to ameliorate disease and fistula healing in patients with CD extraction fistulae. Therefore, adalimumab may not only be as effective as other anti-TNFs but also have fewer anaphylactic and immune reactions [74]. Devint et al., in 2014, compared the effect of combining adalimumab and ciprofloxacin with adalimumab alone in the treatment of CD and coexisting perianal fistulas. Of the 76 patients, 71% of patients treated with adalimumab in combination with ciprofloxacin and 47% of patients treated with adalimumab in combination with placebo experienced a fistula reduction. Due to the fact that in order to close the fistula in CD, combination therapy with adalimumab and ciprofloxacin is more effective than monotherapy with adalimumab [75].

Frontali et al. evaluated the risk factors for failure to repair recurrent RVF in 61 women. RVF repair was successful in 37 patients. The study found no association between drug use by RVF patients before surgery and the success or failure of surgical treatment. Of the 61 patients, 5 patients were treated with corticosteroids, 8 with azathioprine, 12 with biologics and 6 with antibiotics. Of the 37 patients who successfully repaired the RVF, 21 patients received postoperative antibiotics (PAP), and of the 24 patients with surgical failure, only 3 patients received PAP. Therefore, Frontali et al. showed that PAP reduces the risk of failure from 55% to 14% [17]. The benefits of this therapy mainly concern the prevention of perineal infections, which is very important when it comes to the proper healing of postoperative wounds. There is therefore a need for research to investigate whether the use of antibiotics improves wound healing, particularly in the course of RVF repair.

Tacrolimus is an immunosuppressive drug that inhibits the production of IL-2, IL-3, IL-4 and IL-5 by T cells and the proliferation of B cells [76]. In 2003, Sandborn et al. conducted a 10-week study in which patients with CD fistulae received tacrolimus 0.2 mg/kg/day or a placebo. The study showed that oral tacrolimus effectively improves fistula drainage but does not cause fistula remission [77]. However, due to its nephrotoxicity, it should be used in patients resistant to other treatment methods, such as azathioprine or infliximab [78].

Although there are studies evaluating the effect of antibiotic therapy on RVF, to our knowledge there are still no studies that clearly indicate that the administration of antibiotics in the perioperative period will improve fistula repair and prognosis. Clinicians often prescribe antibiotics before or after surgery to prevent infection and inflammation. In the case of RVF, reducing the number of infections may not only improve the outcome of care during surgery but may also affect the healing of the fistula. Therefore, further research should address the effectiveness of perioperative antibiotics for RVF repair and its recovery time. Difficulties arise from complex etiology and the partially opposite effects that we expect from the treatment regimens simultaneously. On one hand, the immunosuppressive effect is expected especially in inflammatory diseases such as CD; on the other, strong antimicrobials are used to prevent infection. Perhaps the implementation of an appropriate antibiotic therapy algorithm will allow for better results in the treatment of fistulas.

### 5.2. Stoma and Stool Features

The protective effect of creating a diversion stoma on the prognosis of RVF is still unproven; however, according to recommendations, it is still the first step to relieve symptoms and inflammation after the onset of RVF [65]. An inverted stoma reduces the pressure gradient between the anus and the vagina, which may contribute to better healing of the fistula after surgery [4].

Zheng et al., in 2017, conducted a study in which out of 24 patients with RVF, 21 women underwent colostomy or ileostomy. Of these 21 women with a stoma, 18 patients had a transverse colostomy, 1 patient had a transverse colostomy with RVF suture and 2 patients had a terminal ileostomy. The patient who underwent a transverse colostomy with RVF suture and 14 patients with a transverse colostomy recovered after the stoma procedure alone. Patients with fecal diversion did not need fistula repair. The authors of the study showed that most RVFs heal after colostomy or ileostomy, with a median repair time of 222 days. Moreover, the study indicated that RVF patients after colostomy or ileostomy do not need additional surgical procedures, such as muscle or tissue transfer flaps [70].

Barugola et al. conducted a study of the effect of the loop stoma on the healing of postoperative RVF in 37 patients, most often after anterior rectal resection. In 19 women, the fistula healed spontaneously within 6 months after fecal diversion. The mean time was 99.7 days. In 18 women, the loop stoma did not heal within 6 months. The study showed that stool drainage with a transverse colostomy improved RVF healing outcomes [79].

A study from 2016 evaluated the effect of diversion stoma on RVF repair in 62 patients. The 26 patients who underwent stoma drainage had larger fistula sizes than patients operated on without stoma drainage. The rate of postoperative complications and fistula recurrence (44%) did not differ between the two groups. Moreover, the hospital stay of patients with a stoma was longer than that of patients without a stoma. The authors of the study therefore suggested that the presence of a diversion stoma does not affect the frequency of fistula recurrence [80]. Considering the fact that stool drainage was performed only in patients with complex RVF, it can be assumed that failure to create a stoma in these patients may result in an even higher rate of complications and recurrences.

In their study, Corte et al. attempted to evaluate the surgical outcomes of patients with RVF to identify factors that increase the likelihood of fistula healing. Of the 79 patients, 67 patients had a reversible stoma during surgery. In total, 286 procedures were performed in all patients, including 152 procedures involving stoma redirection. The RVF repair success rate was 6% (7/134) in patients without a stoma and 32% (49/152) in patients with a stoma. The reason for this difference is the success rate of 5% in intact conservative procedures compared to the 19% success rate in reverse conservative procedures. The presence of a deflecting stoma increased the rate of fistula healing after local procedures. Moreover, the authors of the study suggested that it would be beneficial to use a diverting stoma, if necessary, in combination with another aggressive procedure, gracilis muscle insertion or ante-rectal retraction [81]. In patients with a fistula, stoma placement may indeed be considered in conjunction with another procedure that would increase the likelihood of RVF healing. Already, in a 1992 study, Rex et al. showed that the rate of fistula healing as a result of deflecting colostomy alone was 35.3%, and in combination with endoanal repair it was 62.5% [82].

The American Society of Colon and Rectal Surgeons in the guidelines indicates the beneficial effect of 3–6 months of supplementation with fiber that increases the stool to eliminate inflammation [65]. To the best of the authors’ knowledge, the exact effect of fiber has not been described in the treatment of RVF, and the only available data are regarding its effect on anal fistulas [83]. Dietary fiber actually has an effect on stool volume due to the reduction in the concentration of substances such as bile acid. The stool of patients taking dietary fiber is harder than that of patients not taking it [84]. Moreover, increased fiber intake leads to a decrease in fecal pH [85]. Perhaps a firmer stool consistency may prevent it from entering the RVF and thus prevent infection or inflammation. Therefore, there is a need for clinical trials comparing the effect of fiber supplementation in RVF patients on fistula healing.

In conclusion, the creation of a drainage stoma, especially in the early stages of RVF treatment, can prevent leakage from spreading and increase the likelihood of fistula healing. It also seems important to determine the importance of the stoma for improving the patient’s situation. It seems that in patients with RVF who had the problem of passing stool through the vagina, the creation of a stoma can stop this problem and significantly improve the quality and comfort of everyday life. On the other hand, complications such as hernia or small bowel obstruction may also occur after stoma formation [82]. Therefore, the decision to defecate should be made by the surgeon, and clear guidelines for stoma formation in patients with RVF would greatly facilitate this. It should be also remembered that a high-fiber diet can also have a potentially beneficial effect on the RVF healing, which may be a reason for fiber supplementation by patients. All studies examining the impact of stoma in the treatment of RVF are presented in the Table 3.

## 6. Discussion

Despite the advances in medicine, RVF is still associated with a high rate of failure and recurrence, as well as poor patient well-being. There are many methods of surgical fistula repair, with treatment success rates from 20% to 100% [1]. The high failure rate in RVF treatment outcomes raises the question: what changes should be made to increase the effectiveness of RVF treatment?

First of all, it is necessary to analyze how differences related to the etiology of RVF affect its treatment. The results of studies from 1986 [24] and 1991 [25] indicate a high risk of RVF repair failure in patients after radiotherapy. Moreover, Karp et al. observed that non-obstetric RVF did not heal as well as obstetric RVF [36]. The results of these studies confirm our supposition that the reason for the poorer fistula healing is non-obstetric etiology. The studies included patients with non-obstetric RVF who were typically older, with comorbidities, and postmenopausal. Perhaps the reason for the worse healing of such fistulae is the lower effect of estrogens on the vaginal trophism in patients [30]. Despite the lack of studies on the effect of estrogens on RVF repair, there are studies confirming improvement in the healing of pelvic or palate tissues after administration of these hormones [36]. Karp et al. [16] should be the basis for assessing the effect of estrogen administration in elderly patients with RVF. In addition, non-obstetric etiology, such as CD or radiotherapy, leads to numerous inflammatory changes in tissues, changes in blood flow, and thus ischemia of the tissues involved in the healing of fistulas. Therefore, in the treatment of non-obstetric RVF, more aggressive surgical procedures and the use of antibiotic prophylaxis should be sought, which may be helpful in fistulae in patients with tissue changes and resistance to treatment.

Despite the lack of studies on the effect on RVF, diabetes and hypertension, leading to numerous inflammations and blood flow disorders, seem to be potential risk factors for poor RVF healing [41,42,43,44,86,87]. Despite the fact that these factors are unmodifiable, efforts should be made to stabilize the conditions of patients with RVF who struggle with these diseases. RVF repair surgeries are usually planned well in advance, so it may be possible to reduce diabetes-related inflammation earlier. It also seems necessary to counsel the patient, to whom the possible benefits of earlier preparation for surgery and treatment of the comorbidity should be explained.

Inflammation in the RVF may cause the microflora of the vaginal and anal tissues to be altered or pathogenic [48,49]. Despite the lack of studies on the use of FMT in the treatment of RVF, remodeling of the intestinal flora may be a helpful therapy for fistula repair. The use of this method in patients with CD and internal and enterocutaneous fistulas resulted in closure of the fistula after the use of this technique [58,59]. The results of the studies indicate a remission of CD after about 9 months, as well as a reduction in diarrhea in patients [59]. Perhaps FMT is a salvage therapy for drug-resistant CD by reducing bacterial involvement in inflammation and thus RVF. Taking into account the previously described poor healing of fistulae resulting from CD, the use of FMT in these patients may be a breakthrough in its treatment. Moreover, it seems important to use FMT to reduce diarrhea, and to implement fiber supplementation, which will increase stool volume, thus preventing feces from entering the fistula and the overgrowth of bacterial species [65,84]. Probiotics, in turn, due to their ability to lower glycaemia and cholesterol [55,56], seem to be helpful in patients with RVF and concomitant diabetes or hypertension.

The dominance of intestinal microflora producing biofilm of medium or high concentration in patients with anal fistula lasting more than 6 months in the study by Jaswail et al. leads to the hypothesis that it is bacteria producing biofilm that may be the cause of the poor healing of fistulae [61]. To our knowledge, this aspect has not yet been explored for RVF. Both Jaswail et al. and Kozlovska et al. have suggested that biofilm-forming bacteria determine the chronic development of CAF and anal fistulas. Although these studies have identified a role for bacteria in long-term anal fistulae, they have not determined the exact contribution of biofilm-forming organisms to infection progression. Determining this mechanism in the context of RVF would allow for the development of effective antibiotic prophylaxis. Moreover, it would also allow us to determine the use of electrophoretic degradation of biofilm, which in the case of CAF proved to be effective and destroyed the matrix [60].

Although the creation of a stoma in patients with RVF seems to significantly improve the daily hygiene and comfort of women’s lives, there are still no data on which group of patients will benefit the most from its creation. Barugola et al. suggested that the creation of a loop stoma improves RVF healing outcomes [79]. Corte et al. showed that a diverting stoma created at an early stage of treatment will increase the rate of RVF healing after surgery [81]. Moreover, the same authors suggested that perhaps stoma formation could be performed in conjunction with other RVF treatment, further increasing the likelihood of fistula healing [81]. This is in line with the studies by Rex et al., who reported almost rates of RVF healing twice as fast in patients after simple colostomy with endoanal repair as compared to colostomy alone [88]. Therefore, it is important to determine which group of patients with RVF would benefit the most from stool drainage combined with another surgical procedure. Perhaps such procedures should be introduced especially in patients with non-obstetric RVF, with more comorbidities or with large fistulas located in the upper half of the vagina, which are characterized by poorer healing. Drainage of the stoma, and especially of the loop colostomy, can also help to achieve more effective irrigation, which will minimize changes in the microbiome and therefore improve repair outcomes. This is in line with the research of Barugola et al. [79] where stoma creation prevented other infection-related complications, such as pelvic sepsis.

To the best of our knowledge, however, there are no studies that would overshadow the impact of having a stoma on the psychological well-being of patients with RVF. Admittedly, other studies indicate that fecal diversion itself may be associated with problems such as sexual dysfunction or feelings of depression [89]. Furthermore, a complication of fecal discharge may also be, among others, hernia or obstruction of the small intestine, which require further operations [90]. However, in patients with RVF who are at risk of a reduced quality of life and complications due to vaginal leakage due to fistula, stool redirection may be critical to improving the quality of daily functioning. For patients with RVF, the motivation to consent to create a stoma may be the vision of no problem with leakage of feces through the vagina, which would be a significant improvement in their comfort and daily hygiene. It therefore seems that the stoma will play a role not only in improving healing and RVF repair, but also in ensuring a better quality of life.

However, we should not forget about limitations of our work, which are connected with limitations of the studies described in our review. First, it cannot be overlooked that most studies were retrospective, which makes it difficult to assess preoperative factors affecting RVF repair. Second, the studies were characterized by a small sample size, which led to the poorer verification of predictors, especially in patients with a rare etiology of RVF. In order to better assess the impact of RVF etiology on its healing, studies should be conducted on a larger group of patients with the same etiology. Moreover, many factors, such as the impact of diabetes, hypertension, FMT or probiotics on RVF repair, still remain undescribed. Perhaps the solution will be to create databases of patients with RVF, gathering the data from multiple high-volume centers, which will facilitate the analysis and impact of preoperative factors and medical procedures on fistula healing in the future.

## 7. Conclusions

In order to achieve positive results of RVF treatment, an individualized approach to the patient seems necessary, which will take into account the etiology of the fistula, age and comorbidities. Efforts should be made to stabilize the patient’s condition with comorbidities that may negatively affect blood flow and immune mechanisms, which will interfere with the fistula repair process. In poorly healing RVF, especially in non-obstetrics or in post-menopausal women, the key may be the drainage of feces, the use of antibiotic prophylaxis or the implementation of estrogen therapy. In order to stabilize the composition of the microfilm, which is changed in patients with RVF, hope can be associated with FMT or the use of probiotics. Moreover, it seems necessary to conduct microbiome research in women with RVF and towards estrogen therapy, the results of which may help in the implementation of specific management and treatment algorithms in women with fistulae. The introduction of preoperative interventions may have a positive impact not only on the healing and repair of RVF but also on the comfort and quality of life of patients. The above-mentioned observations suggest that the treatment of RVF should be performed in specialized centers with appropriate experience and access to holistic multidisciplinary treatment coverage apart from surgery and comorbidities control, as well as psychological and lifestyle counseling.

## Figures and Tables

**Table 1:** Studies examining the effect of CD on RVF repair.

The Authors of the Study	The Year of the Study	Research Group	Main Findings
Penninckx et al. [18]	2001	32 patients with contominant CD and RVF.	Analysis identified that Crohn’s sites, presence of extra-intestinal disease and previous Crohn’s proctitis are related with bad healing after a surgical repair.
de la Poza et al. [20]	2012	1215 patients with CD, including 47 patients with fistula and 35 patients with RVF.	No influence of the location and clinical picture of CD on the treatment of genital fistulae was observed.
Narang et al. [19]	2016	99 patients with contominant CD and RVF.	63% rate in RVF healing after surgical repair, no impact of immunomodulation on RVF healing.
Frontali et al. [17]	2021	68 patients with fistula, including 51 patients with RVF and 30 patients with CD.	Age, preoperative treatment and comorbidities do not influence RVF repair failure.

**Table 2:** Studies examining the microbiome in anal fistulas.

Authors of the Study	The Year of the Study	The Number of Patients	Type of Fistula	Main Findings
Leach et al. [48]	2021	14	RVF	They showed that the rectal and vaginal microbiome of patients undergoing successful RVF repair was different from that of patients with RVF recurrence. Patients with successful RVF repair showed the presence of i.a. Bacteroidetes, Alistipes and Rikenellaceae.
Mitalas et al. [50]	2012	44	Transsphincteric fistula of cryptoglandular origin undergoing transrectal lobe repair	They showed epithelialization of the distal and intersphincteric sections of the fistula.
van Onkelen et al. [51]	2013	10	Crypto-nodal transsphincteric fistula undergoing transanal advancement flap procedure	They demonstrated the pro-inflammatory peptidoglycan and the host response to it. The study suggests that peptidoglycan secretes interleukin 1β and other inflammatory mediators in perianal fistulas.
van Onkelen et al. [52]	2016	27	Cryptoglandular transsphincteric fistula who underwent flap repair, sphincterduct ligation or both	They showed that IL-1β is expressed in the majority of anal glandular crypto-fistulas.

**Table 3:** Studies examining the impact of stoma in the treatment of RVF.

Authors	Year	Number of Patients	Type of Stoma	Conclusions
Corte et al. [81]	2015	79	Diverting stoma	Gradual RVF surgical treatment can be changed to more agressive with a diverting stoma and a major surgery.
Lambertz et al. [80]	2016	62	Reversing stoma	They suggested that the presence of a diversion stoma do not influence the recurrence of RVF.
Zheng et al. [70]	2017	24, including 21 who underwent colostomy or ileostomy	18 patients with a transverse colostomy, 1 patient with a transverse colostomy and RVF suture, 2 patients with terminal ileostomy	They suggested that most of RVF can by healed by a diverse stoma, with or without an additional surgery.
Barugola et al. [79]	2021	37	Loop stoma	They suggested that a step-up approach with a loop stoma for at least 6 months can represent changes to a more specific treatment.

## Data Availability

Not applicable.
